# Hydrothermal Synthesis of Co-Doped NiSe_2_ Nanowire for High-Performance Asymmetric Supercapacitors

**DOI:** 10.3390/ma11081468

**Published:** 2018-08-18

**Authors:** Yun Gu, Le-Qing Fan, Jian-Ling Huang, Cheng-Long Geng, Jian-Ming Lin, Miao-Liang Huang, Yun-Fang Huang, Ji-Huai Wu

**Affiliations:** Engineering Research Center of Environment-Friendly Functional Materials, Ministry of Education, College of Materials Science and Engineering, Huaqiao University, Xiamen 361021, China; 1611302006@hqu.edu.cn (Y.G.); 1611302010@hqu.edu.cn (J.-L.H.); 17013081007@hqu.edu.cn (C.-L.G.); jmlin@hqu.edu.cn (J.-M.L.); huangml@hqu.edu.cn (M.-L.H.); huangyf@hqu.edu.cn (Y.-F.H.); jhwu@hqu.edu.cn (J.-H.W.)

**Keywords:** Co-doped NiSe_2_, nanowire, hydrothermal method, pseudocapacitance, asymmetric supercapacitors

## Abstract

Co@NiSe_2_ electrode materials were synthesized via a simple hydrothermal method by using nickel foam in situ as the backbone and subsequently characterized by scanning electron microscopy, transmission electron microscopy, energy-dispersive X-ray spectroscopy, and a specific surface area analyzer. Results show that the Co@NiSe_2_ electrode exhibits a nanowire structure and grows uniformly on the nickel foam base. These features make the electrode show a relatively high specific surface area and electrical conductivity, and thus exhibit excellent electrochemical performance. The obtained electrode has a high specific capacitance of 3167.6 F·g^−1^ at a current density of 1 A·g^−1^. To enlarge the potential window and increase the energy density, an asymmetric supercapacitor was assembled by using a Co@NiSe_2_ electrode and activated carbon acting as positive and negative electrodes, respectively. The prepared asymmetrical supercapacitor functions stably under the potential window of 0–1.6 V. The asymmetric supercapacitor can deliver a high energy density of 50.0 Wh·kg^−1^ at a power density of 779.0 W·kg^−1^. Moreover, the prepared asymmetric supercapacitor exhibits a good rate performance and cycle stability.

## 1. Introduction

Supercapacitors are excellent energy-storage devices which are attracting increased research attention. Although lithium-ion batteries or nickel–metal hydride batteries have a high energy density, their charge and discharge rates are relatively slow and cannot meet the needs of modern life [1]. People are eagerly pursuing energy-storage devices with high energy densities and high power densities simultaneously [2,3]. Compared with traditional batteries, supercapacitors have a high power density, high charge and discharge rates, a wide operating temperature range, environmental protection, and a long cycle life [4,5]. Therefore, they have been widely used in portable electronics, power backup, electric vehicles, various microdevices, and other fields [6].

However, an issue related to the supercapacitors is the lower energy density compared with battery systems or fuel cells [7]. According to the calculation formula for the energy density of the supercapacitor: *E* = 1/2*CV*^2^, the energy density (*E*) can be evaluated by the specific capacitance (*C*_s_) and the cell voltage (*V*). To further increase the energy density, considering both the advantages of supercapacitors (rate, cycle life) and advanced batteries (energy density), assembly of an asymmetric supercapacitor is a commonly used strategy, where the electrode materials with high pseudocapacitance can be employed and the operating voltage can be expanded [8,9]. However, the key to preparing asymmetrical supercapacitors is the selection of suitable positive and negative materials that can function stably under different potential windows in the same electrolyte [10]. This condition is important for the design and preparation of positive and negative materials for asymmetric supercapacitors. Carbon-based materials, such as graphene, activated carbon (AC), carbon fiber, and carbon nanotubes are used as negative materials due to their high electrical conductivity, high specific surface area, good thermal stability, corrosion resistance, controllable pore structure, and other unique physical and chemical properties [11,12,13]. Among them, AC is generally chosen as the negative electrode material of asymmetric supercapacitors due to the advantages of a high specific surface area, low cost, and stable performance [14].

Recently, increasing attention has been paid to transition metal sulfides as they exhibit a higher theoretical capacitance and better electric conductivity than transition metal oxides [15]. The charge storage mechanisms and the electrochemical properties of transition metal sulfides, such as Ni_3_S_4_, CoS, MoS_2_, and CuS, have been widely studied as electrode materials in supercapacitors or asymmetric supercapacitors. However, due to the volume change of sulfide electrode materials during charging and discharging, they are easily detached from the current collectors, which affects their electrochemical performances [16]. Therefore, finding a good material becomes a challenge [17]. Although metal selenide also belongs to the metal chalcogen compounds and can produce a high pseudocapacitance, few reports on the use of metal selenides in supercapacitors are available to date. The published works indicate that selenide has a high theoretical specific capacitance and good electrochemical performance. For example, Wang et al. [18] prepared a cubic NiSe_2_ electrode material under hydrothermal conditions, showing a specific capacitance of 1044 F·g^−1^ at 3 A·g^−1^, Recently, Huang et al. [19] reported a layered MoSe_2_ nanosheet electrode material prepared on a Ni foam substrate by a simple hydrothermal method exhibiting a high specific capacitance of 1114.3 F·g^−1^ at 1 A·g^−1^ and excellent cycle life due to its specific structure. Zhang et al. [20] prepared a unique double-shelled hollow structure CoSe_2_/C composite with heterogeneous intervals between the two shells with a high specific capacitance of 726 F·g^−1^ at 2 A·g^−1^.

To further obtain a selenide electrode material with superior electrochemical performances, we attempt to dope using a pseudocapacitive metal ion, which may lead to a greater abundance in the redox reactions to produce a high capacitance, and an improvement in the electrochemical performances due to the complementary advantages of a doped metal ion and the synergistic effects of the doped metal ion and the host ions.

In this article, Co^2+^ was doped in situ into a NiSe_2_ electrode material (Co@NiSe_2_) grown on Ni foam using a simple hydrothermal method. The prepared Co@NiSe_2_ electrode material has a specific capacitance of 3167.6 F·g^−1^ at a current density of 1 A·g^−1^ in 3 M KOH aqueous solution. Using the Co@NiSe_2_ electrode material as the positive electrode and AC as the negative electrode, an asymmetric supercapacitor was assembled to increase the potential window and the energy density [21,22]. The energy density reaches 50 Wh·kg^−1^ at a power density of 779 W·kg^−1^. Thus, the asymmetric supercapacitor exhibits good electrochemical performance and stable cycle life.

## 2. Experimental Section

### 2.1. Materials

Anhydrous ethanol, acetone, 60% aqueous solution of polytetrafluoroethylene (PTFE), urea (CH_4_N_2_O), and acetylene black were purchased from Sinopharm Group Chemical Reagent Co., Ltd. (Shanghai, China). Cobalt chloride hexahydrate (CoCl_2_·6H_2_O), Nickel chloride hexahydrate (NiCl_2_·6H_2_O), and selenium dioxide (SeO_2_) were available from Aladdin Industrial Corporation (Shanghai, China). Nickel foam was purchased from Changsha Liyuan New Material Company (Changsha, China). AC was purchased from Fuzhou Yihuan Co., Ltd. (Fuzhou, China). All materials are commercially available and do not require further purification.

### 2.2. Preparation of Co@NiSe_2_

To remove the oxide layer and other impurities on the surface of the nickel foam, the nickel foam was sonicated in acetone for 2 h, followed by ultrasonication with anhydrous ethanol for 2 h, repeated washing with distilled water, drying, and allowed to stand. To make the electrode material grow stably and uniformly on the nickel foam, before the reaction, we trimmed the treated nickel foam to 1 × 1 cm^2^ and placed it in a plasma cleaner (PDC-001, Xmcreat, Xiamen, China) for 2 min for secondary treatment.

Here, we synthesized a Co@NiSe_2_ electrode material using a simple hydrothermal method. First, 1 mmol of NiCl_2_·6H_2_O and 2 mmol of SeO_2_ were dissolved in 50 ml of deionized water and stirred until they completely dissolved. Next, 15 mmol CH_4_N_2_O was added and continuously stirred at room temperature for 30 min. Then, a certain amount of CoCl_2_·6H_2_O was added to obtain a uniformly dispersed mixed solution. After the secondary treatment, nickel foam was stably suspended in a Teflon-lined stainless-steel autoclave with a capacity of 100 mL, and the above dispersed mixed solution was transferred into the autoclave. Subsequently, the autoclave was sealed in an electric oven at 180 °C for 16 h. After the hydrothermal process was completed, the nickel foam carrying the active material was taken out and washed several times with distilled water and ethanol. After drying in a vacuum oven at 80 °C for 12 h, a Co@NiSe_2_ electrode material was finally obtained, and the loading of the active material was approximately 2.5 mg·cm^−2^. The samples were named NiSe_2_, Co@NiSe_2_-1, Co@NiSe_2_-2, and Co@NiSe_2_-3 for the addition of 0, 1, 2, and 3 mmol CoCl_2_·6H_2_O, respectively.

### 2.3. Assembly of Asymmetrical Supercapacitors

Here, we used the prepared nickel foam loaded with the Co@NiSe_2_ electrode material as a positive electrode, AC as a negative electrode, and 3 M KOH aqueous solution as an electrolyte to assemble asymmetrical supercapacitors. First, we prepared a negative active carbon by a typical method [23]. AC, acetylene black, and polytetrafluoroethylene were dispersed in an appropriate amount of absolute ethanol at a mass ratio of 8:1:1, and the resulting mixture was sonicated for 2 h and then dried in an oven at 80 °C. Excess ethanol was volatilized off, and the mixture was stirred into gel form. Then, the gel-like AC was pressed into a sheet using a roller press. The appropriate amount of AC was cut according to the required amount, and the cut AC sheet was pressed under pressure onto the treated nickel foam and dried in a vacuum oven at 80 °C for 12 h to obtain an AC negative electrode [24].

The most critical problem in the construction of asymmetric supercapacitors is the active material mass matching of the positive and negative electrodes. Without considering the energy loss due to internal resistance in an ideal state, when the capacitor was charged and discharged, the charge conservation principle was used for the calculation, illustrated as follows [25]:

(1) $$\;q=C_{s}\times \Delta E\times m$$

From Formula (1), the charge storage capacity mainly depends on the mass-specific capacitance of the electrode (*C*_s_), voltage during charge/discharge (Δ*E*), and the active material mass (*m*) of the electrode. To conserve the charge of both positive and negative levels (*q*^+^ = *q*^−^), the mass ratio of positive (*m*^+^) and negative (*m*^−^) electrodes follows Equation (2) [26]:

(2) $$\frac{m_{+}}{m_{-}}=\frac{C_{-}\times \Delta V_{-}}{C_{+}{}_{}\times \Delta V_{+}}$$

### 2.4. Material Characterization

Phase analysis of the prepared samples was performed on an X-ray powder diffractometer (XRD, CuKα radiation, Bruker D8 Advance, Karlsrube, Germany). The microstructure and morphology of the Co@NiSe_2_ electrode material were observed through field emission scanning electron microscopy (FESEM, S-4800, Hitachi, Tokyo, Japan) and transmission electron microscopy (TEM, H-7650, Hitachi, Tokyo, Japan). Using energy-dispersive X-ray spectroscopy (EDS, INCA Energy, Shanghai, China), the elements of the sample were qualitatively analyzed. The specific surface area and pore size distribution of the samples were determined by a fully automatic specific surface area analyzer (BET, Autosorb-iQ, Altanta, GA, USA).

### 2.5. Electrochemical Measurements

The most effective way to measure the performance of the supercapacitor is to test the electrochemical performance of the electrode material. At room temperature, we used the 3 M KOH aqueous solution as the electrolyte, the platinum wire electrode as the counter electrode, the saturated calomel electrode as the reference electrode, and Co@NiSe_2_ on nickel foam was used as the working electrode. Using an electrochemical workstation (CHI760E, Shanghai Chenhua Instrument, Shanghai, China), cyclic voltammetry (CV), galvanostatic charge–discharge (GCD) test, electrochemical impedance spectroscopy (EIS), and cycle life testing were performed on the electrode material.

In general, the capacitance of a supercapacitor reflects the ability to store charge, mainly reflecting the properties of the electrode material. The GCD test was used to calculate the specific capacitance (*C*_s_) of the supercapacitor according to Equation (3) [27]:

(3) Cs=im∫v dtv22|vivf

where *C*_s_ (F g^−1^) is the specific capacitance; *I* (A) is the current during discharge; Δ*t* (s) is the discharge time; *m* (g) is the mass of the active material; and Δ*V* (V) is the potential during the discharge process.

Energy density (*E*) is an indicator of the performance of a supercapacitor and can directly reflect the capacitor’s ability to store charge. The energy density is related to the specific capacitance of the capacitor and the discharge voltage window as illustrated in Formula (4) [28]:

(4) $$\;E=\frac{C_{s}\Delta V^{2}}{7.2}\;$$

The power density (*P*) is another key index that determines the rapid charge and discharge capacity of a capacitor by Formula (5) [29]:

(5) $$\;P=\frac{3600E}{\Delta t}\;$$

## 3. Results and Discussion

### 3.1. Characterization of Electrode Materials

**Figure 1:**
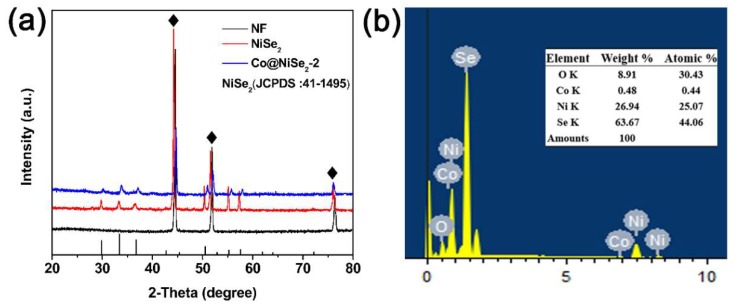
(**a**) X-ray diffraction (XRD) patterns of blank nickel foam (NF), NiSe_2_, and Co@NiSe_2_-2; (**b**) energy-dispersive X-ray spectroscopy (EDS) spectrum of Co@NiSe_2_-2.

To accurately show the structure of the prepared electrode material, we performed an XRD test. Figure 1a shows the blank nickel foam, NiSe_2_, and Co@NiSe_2_-2 electrode materials. The XRD pattern, wherein “♦” represents the three very strong peaks (44.5°, 51.8°, and 76.37°), displays the nickel-foam base (JCPDS no. 04-0850). In addition to the nickel base peaks, the NiSe_2_ diffraction peaks (JPDS no. 41-1495) are 29.79°, 33.40°, 36.70°, 50.48°, and 57.52°, corresponding to the (200), (210), (211), (311), and (230) crystal planes, respectively. In the XRD pattern of Co@NiSe_2_-2, we find a slight shift in the spectrum presumably due to a small amount of Co doping. In addition, the chemical composition of the synthesized product was characterized using EDS analysis, as shown in Figure 1b. The information on Ni, Se, and Co can be seen from the figure, and the surface sample was composed of the above elements. Moreover, the displayed oxygen may come from the oxygen and moisture on the sample surface.

**Figure 2:**
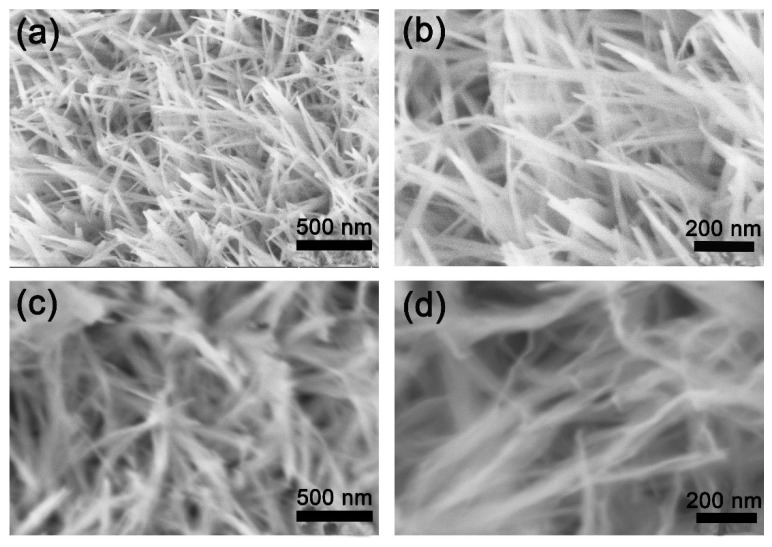

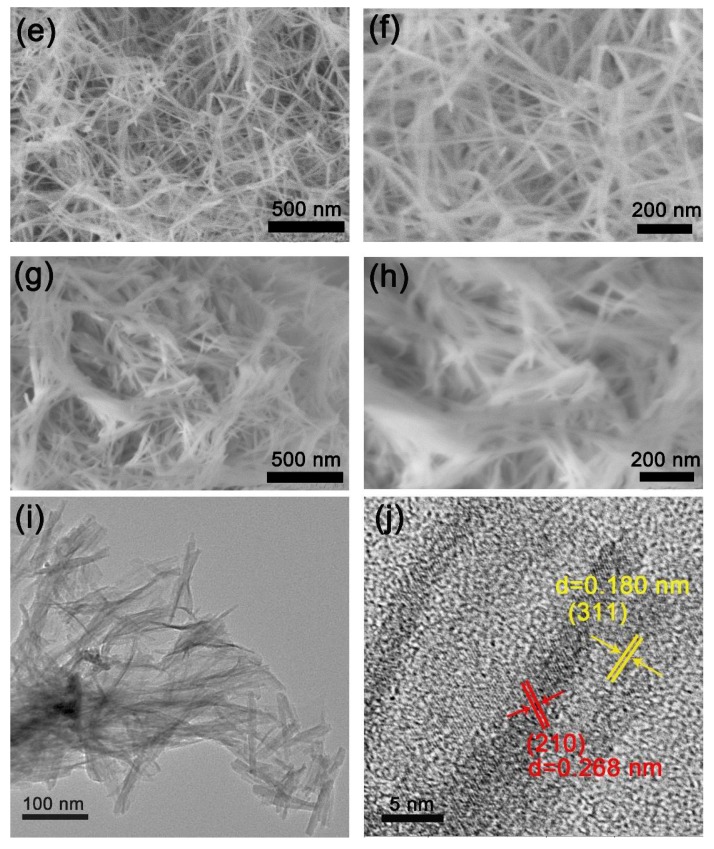
Field emission scanning electron microscopy (FESEM) images of (**a**,**b**) NiSe_2_; (**c**,**d**) Co@NiSe_2_-1; (**e**,**f**) Co@NiSe_2_-2; (**g**,**h**) Co@NiSe_2_-3; (**i**,**j**) transmission electron microscopy (TEM) images of Co@NiSe_2_-2 electrode materials.

Figure 2 shows FESEM images of the NiSe_2_ and Co@NiSe_2_ electrode materials and TEM images of Co@NiSe_2_-2 at different magnifications. As presented in Figure 2a,b, under this hydrothermal condition, a NiSe_2_ nanowire with a width of about 50 nm can be formed. It is observed that it is possible to retain the morphology after doping with Co^2+^, and increased doping with Co^2+^ results in a decreasing width of the wire (Figure 2c–f). However, for Co@NiSe_2_-3, the sample exhibits serious aggregation (Figure 2g–h). Figure 2i also proves that the morphology of Co@NiSe_2_-2 is a nanowire with the width of about 20 nm. Two different crystal faces are seen from the analysis of Figure 2j. The interplanar spacings are *d* = 0.268 nm and *d* = 0.1806 nm, corresponding to the (210) and (311) crystal faces of the NiSe_2_ crystal, respectively. The hydrothermal synthesis of the nanowire structures can greatly increase the specific surface area of the electrode material, effectively increasing the ion transport process and contributing to a higher specific capacitance.

**Figure 3:**
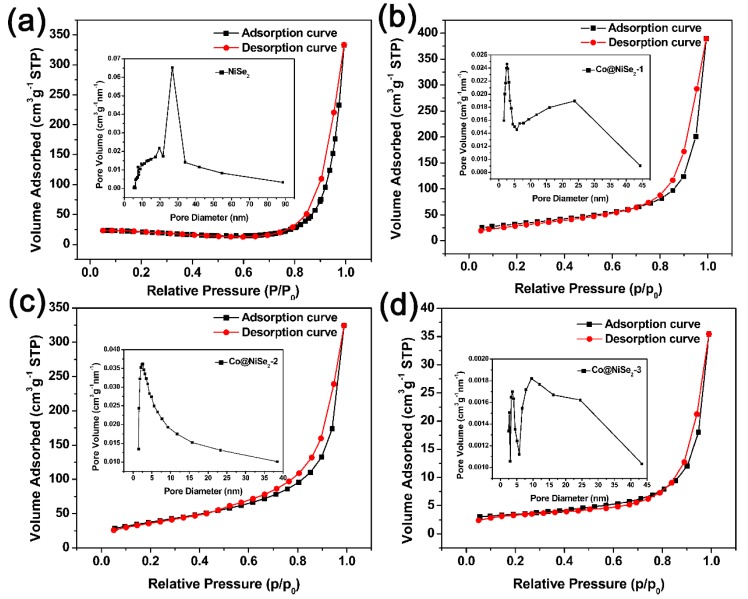
N_2_ absorption–desorption curves and pore size distributions of (**a**) NiSe_2_; (**b**) Co@NiSe_2_-1; (**c**) Co@NiSe_2_-2; and (**d**) Co@NiSe_2_-3 electrode materials.

To study the structure and specific surface area of the nanowires of the electrode material in depth, physical adsorption of nitrogen was used [30]. Figure 3 shows the N_2_ adsorption–desorption curves and pore size distributions of the prepared samples. Their specific surface areas, pore volumes and pore sizes are listed in Table 1. The adsorption–desorption curves of NiSe_2_, Co@NiSe_2_-1, Co@NiSe_2_-2, and Co@NiSe_2_-3 electrode materials all show the IUPAC-H3 hysteresis loop, which is a type of BDDT-IV and belongs to a typical mesoporous structure [31]. The results show that with the increase of the Co^2+^ content of the reactant, the specific surface area increases from 54.6 m^2^·g^−1^ to the maximum value of 135.2 m^2^·g^−1^ (Co@NiSe_2_-2), before decreasing to 11.5 m^2^·g^−1^ (Co@NiSe_2_-3). The reason for the enhancement of the specific surface area can be attributed to the thinner nanowire [32]. However, the aggregation leads to the decrement of the specific surface area for Co@NiSe_2_.

### 3.2. Electrochemical Properties of Electrode Materials

#### Electrochemical Tests of Electrode Materials in a Three-Electrode System

**Figure 4:**
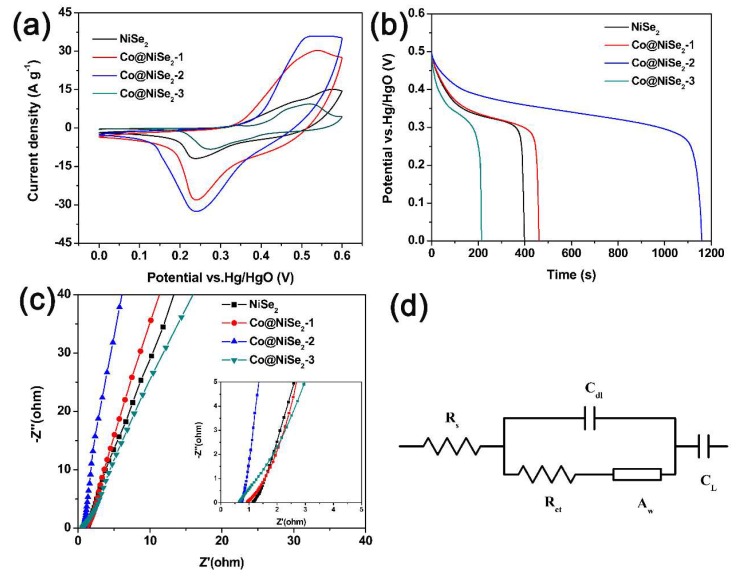
(**a**) Cyclic voltammetry (CV) curves of electrode materials at a scan rate of 5 mV·s^−1^; (**b**) galvanostatic charge-discharge (GCD) curves of electrode materials at a current density of 1 A·g^−1^; (**c**) Nyquist plots of electrode materials (the inset is the enlarged curves at a high-frequency curve range); (**d**) equivalent circuit used to fit the Nyquist spectra.

Figure 4 shows the electrochemical performance of a three-electrode system for NiSe_2_ and Co-doped NiSe_2_ electrode materials in 3 M KOH electrolyte. Figure 4a illustrates their CV curves at a scan rate of 5 mV·s^−1^ and a potential of 0–0.6 V (vs. Hg/HgO). A reversible redox peak is observed in the curves during charge and discharge. This phenomenon illustrates that the NiSe_2_ and Co-doped NiSe_2_ electrode materials show typical pseudocapacitance characteristics, and the CV curve of Co@NiSe_2_-2 surrounds the largest area, indicating that this electrode material can produce the biggest specific capacitance. Figure 4b shows GCD curves of NiSe_2_ and Co-doped NiSe_2_ electrode materials at a current density of 1 A·g^−1^. According to these GCD curves, their specific capacitances are calculated. For NiSe_2_, Co@NiSe_2_-1, Co@NiSe_2_-2, and Co@NiSe_2_-3 electrode materials, the specific capacitances are 1091.3, 1249.6, 3167.6, and 580.4 F·g^−1^, respectively. The enhancement of the specific capacitance after doping with Co^2+^ is possibly due to the increase in the specific surface area of the electrode material and the synergistic effects of Co^2+^ and Ni^2+^. This super high capacitance of the Co@NiSe_2_-2 electrode material is higher than those of the previously reported metal selenide electrodes and some other typical electrode materials, as shown in Table 2.

Figure 4c displays the Nyquist plots of NiSe_2_ and Co-doped NiSe_2_ electrode materials. Impedance testing is a characterization of the dynamic characteristics between the electrode material and the electrolyte [40]. Table 3 shows comparisons of the impedance data of different electrode materials. R_s_ is related to the resistances of the current collector, the electrode material, and the electrolyte. R_ct_ represents the charge transfer resistance. Z_w_ refers to the ion diffusion resistance in the electrolyte. As can be seen from Table 3, Co@NiSe_2_-2 has the smallest R_s_ (0.72 Ω·cm^2^), R_ct_ (0.82 Ω·cm^2^), and Z_w_ (0.0012 Ω·s^−1/2^·cm^2^), which are beneficial; therefore, Co@NiSe_2_-2 exhibits superior electrochemical performances. This indicates that the Co@NiSe2-2 electrode has the best electronic conductivity and the fastest charge transfer rate. Therefore, we chose a Co@NiSe_2_-2 electrode material for the following in-depth study.

**Figure 5:**
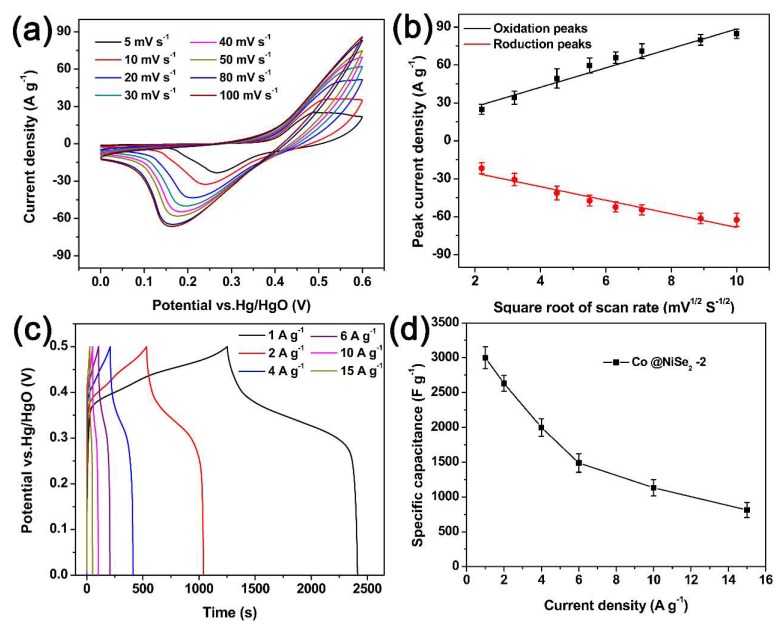
(**a**) CV curves of Co@NiSe_2_-2 electrode at different scan rates; (**b**) relationship of the square root of the scan rate and the peak current; (**c**) GCD curves of Co@NiSe_2_-2 electrode at different current densities; (**d**) specific capacitance of Co@NiSe_2_-2 electrode at different current densities.

Figure 5 describes the electrochemical tests of a Co@NiSe_2_-2 electrode material in a 3 M KOH electrolyte using a three-electrode system. Figure 5a shows the CV curves at different scan rates (5–50 mV·s^−1^) for a Co@NiSe_2_-2 electrode at a potential of 0–0.6 V vs Hg/HgO. The charge and discharge curves show a pair of reversible redox peaks, indicating that the Co@NiSe_2_-2 electrode exhibits typical pseudocapacitance properties. With the increase of the scanning rate, the redox peak reveals a potential shift, but the shape of the curve does not change significantly. The change signifies that the electrode material not only had good reversibility but can also work stably at a potential window of 0–0.6 V vs Hg/HgO [41]. Figure 5b shows the plot of the square root of the scan rate and the peak current. This figure illustrates that both the oxidation peak and the reduction peak have an approximately linear relationship with the square root of the scan rate. This condition demonstrates the redox reaction at the electrolyte/electrode interface, corresponding to quasi reversible and diffusion control processes [42]. Figure 5c shows the GCD curves of the Co@NiSe_2_-2 electrode in a potential window of 0–0.5 V vs Hg/HgO at different current densities. The GCD curves show that each line does not change linearly. Distinct charging and discharging platforms are observed due to reversible oxidation and reduction. Using the integral calculation Formula (3) to calculate the specific capacitances of the Co@NiSe_2_-2 electrodes, the results are 3167.6, 2697.6, 2079.4, 1572.9, 1202.4, and 885.6 F·g^−1^ at current densities of 1, 2, 4, 6, 10, and 15 A·g^−1^, respectively, as shown in Figure 5d. As the current density increases, the capacitance declines, signifying that the kinetics of the redox reaction depend primarily on the diffusion and migration of ions in the electrolyte [43]. The migration and diffusion rates of electrolyte ions were suppressed at high current densities.

After the three-electrode test on the Co@NiSe_2_-2 electrode material, we will use the AC electrode as the negative electrode and the Co@NiSe_2_-2 electrode as the positive electrode to fabricate an asymmetric supercapacitor. The electrochemical performance of the capacitor will be further explored and analyzed.

**Figure 6:**
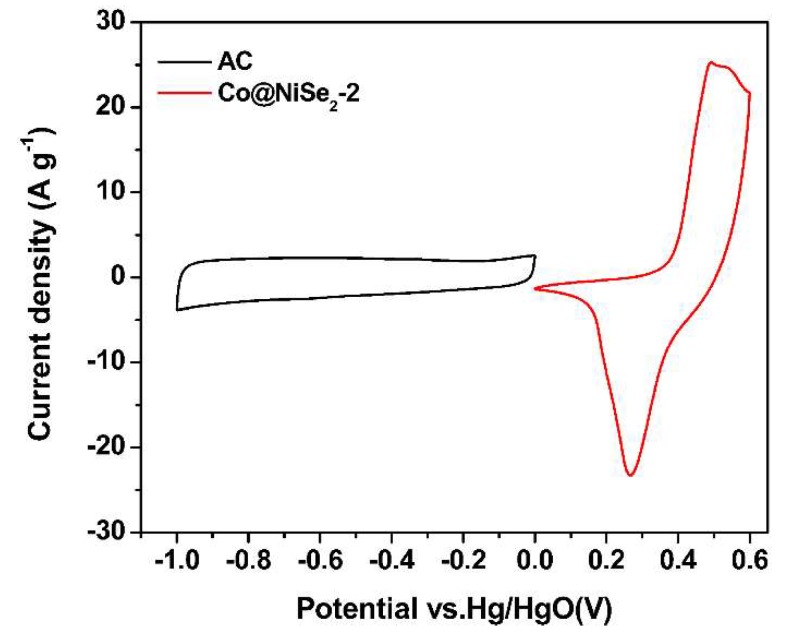
CV curves of the activated carbon (AC) electrode and the Co@NiSe_2_-2 electrode in a three-electrode system.

Figure 6 is a comparison of the CV curves of an AC electrode and a Co@NiSe_2_-2 electrode at a scan rate of 5 mV·s^−1^ in a three-electrode system. As can be seen from the figure, the potential window of the AC electrode is −1–0 V, and the AC electrode exhibits typical double-layer capacitance characteristics, whereas the potential window of the Co@NiSe_2_-2 electrode was 0–0.6 V, showing a satisfying pseudocapacitance performance.

The asymmetric supercapacitor was composed of an AC electrode as the negative electrode and a Co@NiSe_2_-2 electrode as the positive electrode. These two electrodes have a different energy storage mechanism, potential window, and specific capacitance; thus, they must be in accordance with the principle of charge storage [44]. At a current density of 1 A·g^−1^, the specific capacitances of the Co@NiSe_2_-2 electrode and the AC electrode are 3167.6 and 253.6 F·g^−1^, respectively. Therefore, according to Formula (2) for the calculation of the quality of the AC electrode, we determined the best quality ratio of positive and negative materials as approximately m (Co@NiSe_2_-2)/m (AC) = 0.16 and assembled them into an asymmetric supercapacitor for testing.

**Figure 7:**
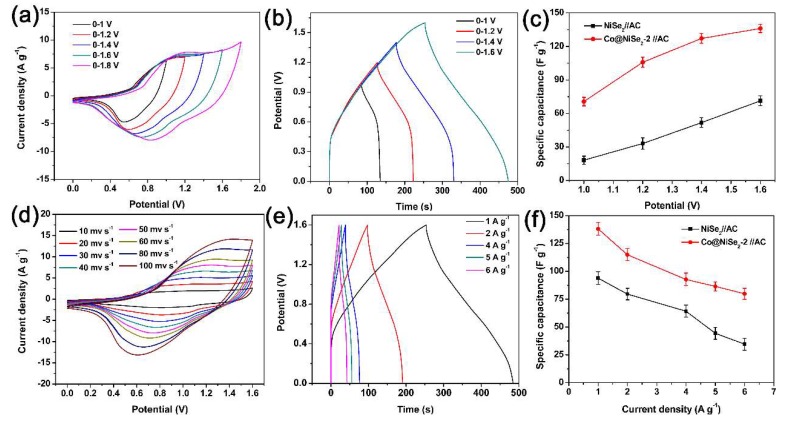
(**a**) CV curves of Co@NiSe_2_-2//AC asymmetric supercapacitor at a scan rate of 50 mV·s^−1^ at different potential windows; (**b**) GCD curves of Co@NiSe_2_-2//AC asymmetric supercapacitor at different potential windows at a current density of 1 A·g^−1^; (**c**) plots of specific capacitances for NiSe_2_//AC and Co@NiSe_2_-2//AC asymmetric supercapacitors at different potential windows; (**d**) CV curves of Co@NiSe_2_-2//AC at different scanning rates; (**e**) GCD curves of Co@NiSe_2_-2//AC asymmetric supercapacitor at different current densities; (**f**) plots of specific capacitances for NiSe_2_//AC, Co@NiSe_2_-2//AC asymmetric supercapacitors at different current densities.

Figure 7a shows the CV curves for the Co@NiSe_2_-2//AC asymmetric supercapacitor at different potential windows and a scan rate of 50 mV·s^−1^. Notably, the CV curve maintains a similar shape in the potential window of 1 to 1.6 V. When the potential window extends to 1.8 V, a polarized phenomenon appears. Thus, we can confirm that the asymmetric supercapacitor can be stabilized in the potential window of 0 to 1.6 V. From the analysis results of the CV curves, we specify the subsequent test potential window from 0 to 1.6 V. Figure 7b shows the GCD curves of the Co@NiSe_2_-2//AC asymmetric supercapacitor at a current density of 1 A·g^−1^ under different potential windows. As can be seen from the figure, all curves show exceptional consistency once again. The results show that the Co@NiSe_2_-2 electrode has excellent electrochemical performance and stability. Figure 7c reveals the plots of specific capacitances for NiSe_2_//AC and Co@NiSe_2_-2//AC asymmetric supercapacitors at different potential windows. For both asymmetric supercapacitors, the specific capacitances increase with the increment of potential window. For the Co@NiSe_2_-2//AC asymmetric supercapacitor, when the potential window is from 1 to 1.6 V, the specific capacitance is enhanced from 70.64 to 136.06 F·g^−1^. Both the original and enhanced specific capacitances of the Co@NiSe_2_-2//AC asymmetric supercapacitor are much higher than those of NiSe_2_//AC (from 18.16 to 71.31 F·g^−1^). Figure 7d shows CV curves of Co@NiSe_2_-2//AC at different scanning rates of 10–100 mV·s^−1^ and with the same potential window of 0–1.6 V. No significant deformation of the CV curve is observed as the scan rate increases. In addition, each curve presents a similar quasi-rectangular shape, indicating the cooperative behavior of pseudocapacitive Co@NiSe_2_-2 electrode and the electric double-layer capacitive AC electrode. In addition, this phenomenon indicates that this asymmetric supercapacitor has rapid charge–discharge reversibility. Figure 7e shows GCD curves of the Co@NiSe_2_-2//AC asymmetric supercapacitor at different current densities. The specific capacitances at current densities of 1, 2, 4, 5, and 6 A·g^−1^ are 140.6, 121.2, 98, 88.7, and 80.6 F·g^−1^, respectively. The specific capacitance for the Co@NiSe_2_-2//AC asymmetric supercapacitor at 6 A·g^−1^ is 57.3% of that at 1 A·g^−1^ in the case of a sixfold increase in the current density, which is higher than that for the NiSe_2_//AC asymmetric supercapacitor (38.9%), demonstrating that the assembled Co@NiSe_2_-2//AC asymmetric supercapacitor presents a good rate performance, as shown in Figure 7f.

**Figure 8:**
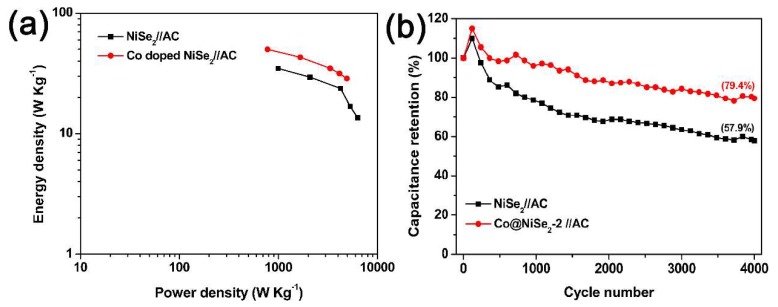
(**a**) Ragone polts; (**b**) cyclic performances tested at a current density of 1 A·g^−1^ of NiSe_2_//AC and Co@NiSe_2_-2//AC asymmetric supercapacitors.

Figure 8a shows the Ragone plots of NiSe_2_//AC and Co@NiSe_2_-2//AC asymmetric supercapacitors, which refer to the relationship of energy density and power density derived from GCD curves at different current densities. The Co@NiSe_2_-2//AC asymmetric supercapacitor can reach the maximum energy density of 50 Wh·kg^−1^ at a power density of 779 W·kg^−1^, which exceeds that of the NiSe_2_//AC asymmetric supercapacitor (34.8 Wh·kg^−1^ at 998 W·kg^−1^), and those of most previously reported NiSe_2_-based supercapacitors [18,19,20]. Figure 8b shows the cycle life for NiSe_2_//AC and Co@NiSe_2_-2//AC asymmetric supercapacitors over 4000 charge-discharge cycles at a constant current density of 1 A·g^−1^. Both capacitors exhibit an increase in capacitance in the first 200 cycles, which is assigned to the activation process [45], and then present a decrease in capacitance. After 4000 cycles, the capacitance retention rate for the Co@NiSe_2_-2//AC asymmetric supercapacitor is 79.4%, which is higher than that of the NiSe_2_//AC asymmetric supercapacitor (57.9%), indicating good cycling stability.

The possible reasons for the superior electrochemical performances of Co@NiSe_2_-2//AC asymmetric supercapacitor are shown as follows: 1) This unique nanowire structure is beneficial to the transfer of electrons; 2) the doping of Co^2+^ into NiSe_2_ can increase the specific surface area, leading to an increase in the use of active material; 3) the direct growth of the electrode material onto the current collector (Ni foam) does not require extra binder and conductive additive, avoiding a decline in the electrochemical performance caused by obstruction of the hole in the electrode material.

## 4. Conclusions

The Co@NiSe_2_ electrode material with a nanowire structure was synthesized by a simple one-step hydrothermal method with nickel foam as the supporter. Proper doping of Co^2+^ into NiSe_2_ can greatly improve the electrochemical performances of electrode material. The specific capacitance of the Co@NiSe_2_-2 electrode can reach up to 3167.6 F·g^−1^ at the current density of 1 A·g^−1^. This Co@NiSe_2_-2 electrode as the positive electrode assembled with AC as the negative electrode lead to a Co@NiSe_2_-2//AC asymmetric supercapacitor. The asymmetric supercapacitor can reversibly work in the potential window of 0‒1.6 V, shows a high energy density of 50 Wh·kg^−1^ at a power density of 779 W·kg^−1^. In addition, after 4000 charge-discharge cycles, a retention rate of 79.4% can be achieved. The results show that the obtained Co@NiSe_2_-2 electrode material has potential as a supercapacitor electrode material.

## Figures and Tables

**Table 1:** Comparisons of surface area analysis of different electrode materials.

Sample	BET (m^2^·g^−1^)	Pore Volume (cm^3^·g^−1^)	Pore Size (nm)
NiSe2	54.6	0.5	>30
Co@NiSe_2_-1	115.7	0.6	>25
Co@NiSe_2_-2	135.2	0.5	>5
Co@NiSe_2_-3	11.5	0.05	>25

**Table 2:** Comparisons of supercapacitor performances with past typical materials.

Electrode Material	Electrolyte	Morphology Structure	Specific Capacitance (F·g^−1^)	Ref.
Co_3_O_4_/rGO	2 M KOH	Cubic morphology	487 F·g^−1^ at 5 mV·s^−1^	[33]
NiO	2 M KOH	Nanosheet hollow spheres	600 F·g^−1^ at 10 A·g^−1^	[34]
PANI/MoS_X_	1 M H_2_SO_4_	Nanoparticle	48.64 mF·cm^−2^ at 56.62 μA·cm^−2^	[35]
GO/PPy	1 M Na_2_SO_4_	Nanoparticle	332.6 F·g^−1^ at 0.25 A·g^−1^	[36]
NiS	3 M KOH	Microflower	1122.7 F·g^−1^ at 1 A·g^−1^	[37]
CoS	6 M KOH	Nanotubes	285 F·g^−1^ at 0.5 A·g^−1^	[38]
NiTe	3 M KOH	Nanorods	804 F·g^−1^ at 1 A·g^−1^	[39]
CoSe/C	2 M KOH	Nanoparticles	726 F·g^−1^ at 1 A·g^−1^	[20]
NiSe_2_	4 M KOH	Nano cube	1044 F·g^−1^ at 3 A·g^−1^	[18]
MoSe_2_	6 M KOH	Nanosheets	1114.3 F·g^−1^ at 1 A·g^−1^	[19]
Co-doped NiSe_2_	3 M KOH	Nanowire	3167.6 F·g^−1^ at 1 A·g^−1^	This work

**Table 3:** Comparisons of impedance data of different electrode materials.

Sample	R_s_ (Ω·cm^2^)	R_ct_ (Ω·cm^2^)	Z_w_ (Ω·s^−1/2^·cm^2^)
NiS_e2_	1.14	1.45	0.0013
Co@NiSe_2_-1	0.93	1.48	0.0026
Co@NiSe_2_-2	0.72	0.82	0.0012
Co@NiSe_2_-3	0.86	1.11	0.0015
